# Automatic PID Control Strategy via Energy Dissipation for Tapping Mode Atomic Force Microscopy

**DOI:** 10.3390/s25144277

**Published:** 2025-07-09

**Authors:** Yuan Zhao, Sha-Sha Xiao, Ji-Rui Liu, Sen Wu

**Affiliations:** State Key Laboratory of Precision Measurement Technology and Instruments, Tianjin University, Tianjin 300072, China; 1023202072@tju.edu.cn (Y.Z.); shashaxiao@tju.edu.cn (S.-S.X.); 1020202043@tju.edu.cn (J.-R.L.)

**Keywords:** tapping-mode atomic force microscopy, automatic PID control, phase lag, energy dissipation, SIMULINK

## Abstract

This study presents an automatic PID control strategy for Tapping-Mode Atomic Force Microscopy (TM-AFM) that addresses the impacts of energy dissipation on tip–sample interactions. Our methodology integrates energy analysis to quantify the critical relationship between energy loss and phase lag dynamics in tapping mode. Additionally, systematic decomposition of interaction force is performed to enable the reconstruction of system transfer functions. The study in this work examines the fluctuations of PID gains during critical oscillations. A SIMULINK-based virtual TM-AFM is developed to simulate practical measurement conditions, based on which a lookup table for PID gains across various phase lags is generated. The efficacy of the proposed algorithm is experimentally validated through the experiments of a calibration nanogrid and two kinds of coated silicon samples, demonstrating the improved tracking accuracy and the improvement of surface height of 5.4% compared to regular control scheme.

## 1. Introduction

Scanning probe microscopy (SPM) has become a milestone of nanotechnology due to its capability to resolve nanoscale topography and spatially map physicochemical properties. Central to SPM operation is the localized interaction between a probe and the sample surface, enabling quantitative extraction of both morphological features and material characteristics with atomic-level resolution [1]. The versatility of SPM is reflected in its diversified modalities, systematically categorized into Atomic Force Microscopy (AFM) for mechanical probing [2], Scanning Tunneling Microscopy (STM) for electronic state analysis [3] and Scanning Near-Field Optical Microscopy (SNOM) for near-field optical imaging [4], etc., each optimized for specific measurement scenarios through distinct tip–sample coupling mechanisms.

Among the operational modes of AFM, Tapping Mode AFM (TM-AFM) has been extensively adopted as a standard for high-resolution imaging [5]. In TM-AFM, the cantilever is oscillated near its resonant frequency with controlled amplitude, while a feedback loop maintains constant oscillation amplitude by maintaining constant tip–sample separation [6,7]. This intermittent-contact strategy strikes a critical balance between spatial resolution and minimized sample damage [8]. The operational principle of TM-AFM involves three interconnected stages: cantilever vibration through resonance excitation, cantilever amplitude detection [9] and feedback-controlled positioning based on Proportional–Integral–Derivative (PID) algorithms [10].

Maintaining precise tip–sample tracking necessitates sophisticated tuning of the PID controller to stabilize cantilever oscillations [11]. In industry, state-of-the-art AFMs predominantly rely on manual PID gains adjustment, a process inherently limited by its susceptibility to operator expertise and time-intensive nature [12]. Further complicating this challenge is the dynamics of tip–sample interactions, where different surface properties such as surface structure [13] or surface energy [14] would induce nonlinear shifts in system response. It is challenging to promptly adjust the PID gains when the surface properties of the sample undergo rapid changes during a single measurement [15].

Traditional PID tuning strategies in AFMs predominantly rely on the linear system models with well-defined transfer functions. Empirical methods like Ziegler–Nichols [16], Cohen–Coon [17] and pole placement optimization method [18], etc., often require an a priori system model to perform effective tracking. These traditional approaches face challenges in adapting to varied material properties, as they are inherently based on the static assumptions [19]. An evolution within this framework is relay-based auto-tuning, which utilizes limit cycle oscillations to identify transfer functions for homogeneous surfaces during pre-scans [20]. However, it still falls short when confronted with abrupt material or structural transitions of sample, demanding real-time parameter adjustments. To address these limitations, the fuzzy logic systems have been proposed [21]. By discretizing tracking errors into linguistic variables through Gaussian membership functions, these systems enable rule-based PID gains update without requiring explicit transfer function models [22]. Similarly to the fuzzy control, a control approach that uses lateral scan speed as a feedback can improve tracking accuracy for high-speed AFM [23]. Nevertheless, even such error-based or velocity-based control, which indeed improved the performance of AFMs, remains rooted in the concept of a fixed transfer function [24]. When the interaction between the sample and the AFM tip changes, so does the transfer function, making it difficult to stably and precisely measure sample profiles. More recently, artificial intelligence (AI)-based approaches have been proposed to utilize convolutional neural networks to predict controller gains with high accuracy, and the response velocity can be dynamically adjusted by means of reinforcement learning [25,26]. Yet, these advancements remain relatively weak in terms of theoretical aspects, raising questions about their stability. Ultimately, to fully harness the potential of AFM, in-depth research into the varying properties of samples and the stability of the system is imperative.

Our work addresses these limitations through a phase-lag-based dynamic PID control framework for TM-AFM. Key innovations stem from two pivotal observations: tip–sample adhesion modifies system energy dissipation, manifested as measurable phase lag variation between cantilever displacement and driving signals; critical oscillation thresholds correlate with both PI gains and phase lags. By reconciling energy conservation principles with tip–sample interaction force decomposition, we derive analytical expressions linking the variation in phase lag to the adjustments of PID gains. A SIMULINK-based AFM which was validated through experiments is applied to analyze the effectiveness of the proposed method. A lookup table via phase lag variations is also derived for experiments. The necessity of the proposed method is verified through the experiments of a calibration nanogrid as well as the coated samples.

## 2. Methodology

### 2.1. Principle of TM-AFM

**Figure 1:**
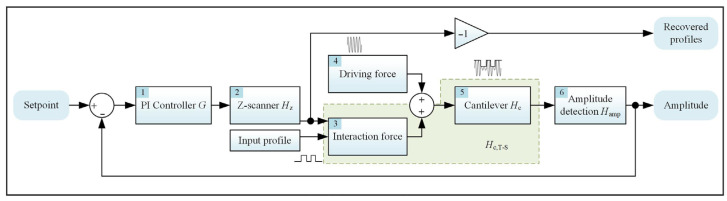
Block diagram of TM-AFM.

Figure 1 depicts the fundamental mechanism of TM-AFM. During the scanning process, the cantilever is excited by the driving force to undergo oscillations close to its resonance frequency. The amplitude of the cantilever’s oscillation is demodulated through the amplitude detection methods, such as the Lock-in Amplifier (LIA) method [27] or the Root Mean Square to Direct Current (RMS-DC) method [28], etc. As the surface height varies during scanning, it results in the amplitude changes due to the interaction force between the tip and the sample. To counteract these variations, a feedback loop is implemented to stabilize the amplitude and relay control signals to the Z-scanner. The morphology of the sample is then retrieved as the negative movements of the Z-scanner. Both the cantilever and Z-scanner can be regarded as second-order underdamped systems. The input voltage of the Z-scanner module is converted to the vertical motions of the cantilever. Taking the cantilever as example, the corresponding dynamics can be described using point-mass model, as shown in Equation (1),

(1) $$m{\ddot{z}}_{c}(t)+c{\dot{z}}_{c}(t)+kz_{c}(t)=F_{\mathrm{drv}}(t)$$

where *m*, *c* and *k* refer to the equivalent masses, damping and elasticities of the cantilever, *F*_drv_(*t*) = *F*_0_cos(*ωt*) is the driving force with *F*_0_ as the amplitude and *ω* is the driving angular frequency. The dynamics of the cantilever can then be solved as Equation (2),

(2) $$\left\{\begin{array}{l} z_{c}(t)=Ce^{\frac{\omega_{0}t}{2Q}}\cos\left[\omega_{0}\sqrt{1-{\left(\frac{c}{4m\omega }\right)}^{2}}t+\psi \right]+\frac{\frac{F_{0}}{m}}{\sqrt{{\left(\omega_{0}-\omega \right)}^{2}+\frac{k^{2}}{c^{2}}\omega^{2}}}\cos(\omega t-\varphi )\\ \varphi =\arctan\left[\frac{\omega_{0}\omega }{\omega_{0}^{2}-\omega^{2}}\frac{1}{Q}\right] \end{array}\right.$$

where coefficient *C* and *ψ* are determined by initial condition, *φ* refers to the phase difference, $Q=\sqrt{mk}/c$ is defined as the quality factor of the cantilever and $\omega_{0}=\sqrt{k/m}$ is the resonant angular frequency of the cantilever. According to Equation (2), if *ω* is equal to the resonant frequency of the cantilever, *φ* without the impact of interaction force would be stabilized at 90°, and the free oscillation amplitude of the cantilever can be derived as *A*_0_ = *F*_0_*Q*/*k*. The transfer functions of the cantilever and the Z-scanner can be outlined in Equation (3),

(3) $$\left\{\begin{array}{l} H_{c}(s)=\frac{1}{ms^{2}+cs+k}\\ H_{z}(s)=\frac{K_{E\text{-}D}\omega_{z}^{2}}{s^{2}+\left(\omega_{z}/Q_{z}\right)s+\omega_{z}^{2}} \end{array}\right.$$

where *s* is the complex frequency of the amplitude errors. *ω*_z_ and *Q*_z_ refer to the resonance angular frequency and quality factor of Z-scanner, and *K*_E-D_ corresponds to voltage-displacement conversion factor. Generally, in the time domain, the transfer function of the amplitude detection module *h*_amp_(*t*) can be treated as convolution of the sine function with a low-pass filter. The PID controller is applied to control the input of the system according to the errors, where *D* gain is usually set as 0 in TM-AFM [29]. Here we define *H*_amp_(*s*) as the Laplace transform of *h*_amp_(*t*) and *G*(*s*) as the transform function of the PID controller. *H*_amp_(*s*) and *G*(*s*) can be formulated as Equation (4),

(4) $$\left\{\begin{array}{l} H_{\mathrm{amp}}(s)=\frac{s}{s^{2}+\omega^{2}}\cdot \frac{\omega_{\mathrm{LP}}}{\omega_{\mathrm{LP}}+s}\\ G(s)=K_{p}+\frac{K_{i}}{s} \end{array}\right.$$

where *ω*_LP_ refers to the cutoff frequency of the low pass filter; *i* and *K_i_* are the corresponding gains of PI controller. Here we define *F*_spec_(*s*) as the spectrum of *F*_drv_(*t*) and *H*_T-S_(*s*) as the transfer function of the interaction module. From Figure 1, it can be observed that both the input profile and the output motions of the Z-scanner module will change the relative position between the tip and the sample, thereby altering the relative force. The displacement caused by the interaction force module will be reflected in the envelope of the motion signal of the cantilever, while the rest caused by the driving force can be equivalent to the carrier signal with relatively higher frequency. The amplitude detection module is employed to extract the envelope of the motions of the cantilever. Therefore, it can eliminate the carrier of the driving signal but will not affect the displacement caused by the interaction force module. The open transfer function of the system can be approximated as Equation (5),

(5) $$\begin{array}{c} TF_{\mathrm{sys}}=\left(GH_{z}H_{T\text{-}S}+F_{\mathrm{spec}}\right)H_{c}H_{\mathrm{amp}}\\ \approx GH_{z}H_{T\text{-}S}H_{c} \end{array}$$

where *TF*_sys_ is the open loop transfer function of the system. Define *H*_c,T-S_ = *H*_T-S_*H*_c_ to express the transfer function of cantilever under the impact of the interaction force; the transfer function of the system can then be reformulated as Equation (6),

(6) $$\left\{\begin{array}{l} TF_{\mathrm{sys}}=GH_{z}H_{c,T\text{-}S}\\ TF_{\mathrm{sys},\;\mathrm{close}}=\frac{GH_{z}H_{c,T\text{-}S}}{1+GH_{z}H_{c,T\text{-}S}} \end{array}\right.$$

where *TF*_sys, close_ refers to the closed loop transfer function of system.

### 2.2. Phase Lag Modeling

**Figure 2:**
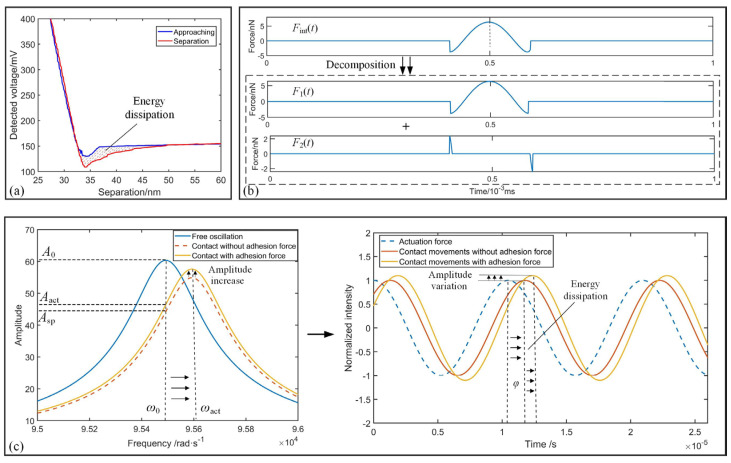
Analysis of energy dissipation to the cantilever. (**a**) Retrieved load-unload curves in TM-AFM. (**b**) Decomposition of interaction force in one movement cycle of the cantilever. (**c**) Impacts of energy dissipation to spectrum and phase lag *φ* in TM-AFM when *φ* falls in 0–90°.

As displayed by the load–unload curves in Figure 2a, affected by the inelastic interactions between the tip and the sample, a greater displacement is required to restore the deflection of the cantilever probe to its initial state during separation. The area enclosed between the load-unload curves would be the energy dissipated, which would impact cantilever dynamics. According to the transfer function in Equation (1), the dynamics of the cantilever considering interaction force can be redescribed through Equation (7),

(7) $$m{\ddot{z}}_{c}(t)+c{\dot{z}}_{c}(t)+kz_{c}(t)=F_{\mathrm{drv}}(t)+F_{T\text{-}S}(t)$$

where *F*_T-S_(*t*) is defined as the interaction force between the tip and the sample. Consequently, the solution in Equation (2) is no longer accurate for the cantilever dynamics, which necessitates considering the effects of energy dissipation. From an energy-conservation standpoint, the power of the driving force acting on the cantilever, in conjunction with its inherent energy losses, must balance the power of energy dissipation. To this end, we introduce *P*_drv_*, P*_dmp-air_ and *P*_T-S_ to represent the power of the driving force, the damping of cantilever in air and the interaction force between the tip and the sample, respectively, as described in Equation (8),

(8) $$\left\{\begin{array}{l} P_{\mathrm{drv}}=F_{\mathrm{drv}}(t){\dot{z}}_{c}(t)\\ P_{\mathrm{damp}\text{-}\mathrm{air}}=-c{\dot{z}}_{c}^{2}(t)\\ P_{T\text{-}S}=F_{T\text{-}S}(t){\dot{z}}_{c}(t) \end{array}\right.$$

Defining *A*_sp_ as the setpoint amplitude, the motion of the cantilever can be expressed as *A*_sp_cos(*ωt* + *φ*), then the averaged power in one movement cycle can be written as Equation (9).

(9) $$\left\{\begin{array}{l} {\bar{P}}_{\mathrm{drv}}=\langle F_{0}\cdot {\dot{z}}_{c}\rangle =\frac{1}{2}F_{0}A_{\mathrm{sp}}\omega \sin\varphi \\ {\bar{P}}_{\mathrm{damp},\;\mathrm{air}}=\langle -c{\dot{z}}_{c}^{2}\rangle =-\frac{1}{2}cA_{\mathrm{sp}}^{2}\omega^{2}\\ {\bar{P}}_{T\text{-}S}=\langle F_{T\text{-}S}\cdot {\dot{z}}_{c}\rangle \end{array}\right.$$

where $\langle \cdot \rangle$ denotes integration operation. Based on Equation (9), we can obtain the expression of ${\bar{P}}_{T\text{-}S}$ as given by Equation (10).

(10) $${\bar{P}}_{T\text{-}S}=-{\bar{P}}_{\mathrm{drv}}-{\bar{P}}_{\mathrm{damp},\;\mathrm{air}}=-\frac{1}{2}\frac{kA_{\mathrm{sp}}^{2}\omega }{Q}\left[\frac{A_{0}}{A_{\mathrm{sp}}}\sin\varphi -\frac{\omega }{\omega_{0}}\right]$$

In addition, in terms of the kinetic energy, the averaged kinetic energy of the cantilever is equal to its virial, so the phase lag *φ* can be further expressed by *F*_T-S_(*t*) based on the virial theory [30], as given by Equation (11),

(11) $${\bar{E}}_{\mathrm{kin}}=\frac{1}{2}m\langle {\dot{z}}_{c}^{2}\rangle =-\frac{1}{2}\left[-k\langle z_{c}^{2}\rangle +\langle F_{\mathrm{drv}}\cdot z_{c}\rangle +\langle F_{T\text{-}S}\cdot z_{c}\rangle \right]$$

where ${\bar{E}}_{\mathrm{kin}}$ denotes the averaged kinetic energy of cantilever. According to Equation (10) and Equation (11), the phase lag *φ* can be expressed as given by Equation (12).

(12) $$\left\{\begin{array}{l} \sin\varphi =\frac{A_{\mathrm{sp}}}{A_{0}}\frac{\omega }{\omega_{0}}\left[1-\frac{2Q\omega_{0}}{kA_{sp}A_{0}\omega^{2}}{\bar{P}}_{T\text{-}S}\right]\\ \cos\varphi \approx -\frac{2Q\langle F_{T\text{-}S}\cdot z_{c}\rangle }{kA_{\mathrm{sp}}A_{0}} \end{array}\right.$$

Energy dissipation is inherently irreversible, thus ${\bar{P}}_{T\text{-}S}$ results in a negative impact. Based on the analysis provided, it can be deduced that the phase lag will increase with the increase in energy dissipation during the contact between the tip and the sample.

### 2.3. System Stability Analysis

To analyze the effects of *F*_T-S_(*t*) on the system stability, an effective approach involves decomposing based on its characteristics. Temporally localize the kinematic trajectory at its nadir, the motion of the cantilever exhibits symmetry on both its left and right sides, while the velocity displays central symmetry throughout one cycle. Motivated by this observation, *F*_T-S_(*t*) can be decomposed into two sub-forces, *F*_1_(*t*) and *F*_2_(*t*), where *F*_1_(*t*) conforms to odd functional components and *F*_2_(*t*) conforms to an even functional components, respectively, as illustrated in Figure 2b. Equation (13) provides the corresponding expressions for *F*_1_(*t*) and *F*_2_(*t*),

(13) $$F_{T\text{-}S}(t)=F_{1}(t)+F_{2}(t)=c_{1}(t)z_{c}(t)+c_{2}(t){\dot{z}}_{c}(t)$$

where *c*_1_(*t*) and *c*_2_(*t*) refer to the coefficients which both accord with even functions. According to Equation (11), $F_{1}(t)\dot{z}(t)$ and *F*_2_(*t*)*z*(*t*) would exhibit odd function distributions and do not generate energy dissipation in one cycle. sin*φ* and cos*φ* can then be redescribed as Equation (14),

(14) $$\left\{\begin{array}{l} \sin\varphi =\frac{A_{\mathrm{sp}}}{A_{0}}\frac{\omega }{\omega_{0}}\left[1-\frac{2Q\omega_{0}}{kA_{sp}A_{0}\omega^{2}}{\bar{P}}_{T\text{-}S}\right]=\frac{A_{\mathrm{sp}}}{A_{0}}\frac{\omega }{\omega_{0}}\left[1-\frac{2Q\omega_{0}}{kA_{sp}A_{0}\omega^{2}}\langle c_{2}\cdot {\dot{z}}_{c}^{2}\rangle \right]\\ \cos\varphi =-\frac{2Q}{kA_{\mathrm{sp}}A_{0}}\langle F_{1}\cdot z_{c}\rangle =-\frac{2Q}{kA_{\mathrm{sp}}A_{0}}\langle c_{1}\cdot z_{c}^{2}\rangle \end{array}\right.$$

Here we define $\bar{c_{1}}$ and $\bar{c_{2}}$ to express the averaged impacts of *F*_int_(*t*) in one cycle, as shown in Equation (15),

(15) $$\left\{\begin{array}{l} {\bar{c}}_{1}\langle z_{c}^{2}\rangle =\langle c_{1}\cdot z_{c}^{2}\rangle \\ {\bar{c}}_{2}\langle {\dot{z}}_{c}^{2}\rangle =\langle c_{2}\cdot {\dot{z}}_{c}^{2}\rangle \end{array}\right.$$

From Equation (15), it would be clear that the sign of $\bar{c_{1}}$ and $\bar{c_{2}}$ accord with $\langle c_{1}\cdot z^{2}\rangle$ and $\langle c_{2}\cdot {\dot{z}}^{2}\rangle$. Compared with Equation (12), since the term ${\bar{P}}_{T\text{-}S}$ results in a negative impact, ${\bar{c}}_{2}$ would be negative accordingly. After that, based on Equation (9) and Equation (11), we can deduce the averaged damping power and the averaged kinetic energy under the impacts of interaction force as Equation (16),

(16) $$\left\{\begin{array}{l} {\bar{P}}_{\mathrm{damp}\_\mathrm{equi}}={\bar{P}}_{\mathrm{damp},\;\mathrm{air}}+{\bar{P}}_{T\text{-}S}=-\left(c+{\bar{c}}_{2}\right)\langle {\dot{z}}_{c}^{2}\rangle \\ {\bar{E}}_{\mathrm{kin}\_\mathrm{equi}}=-\frac{1}{2}\left[-\left(k-{\bar{c}}_{1}\right)\langle z_{c}^{2}\rangle +\langle F_{drv}\cdot z_{c}\rangle \right] \end{array}\right.$$

where ${\bar{P}}_{\mathrm{damp}\_\mathrm{equi}}$ and ${\bar{E}}_{\mathrm{kin}\_\mathrm{equi}}$ denote the averaged damping power and the averaged kinetic energy, respectively. The point mass model in Equation (7) can be reformulated as Equation (17),

(17) $$m\ddot{z}(t)+\left(c+{\bar{c}}_{2}\right)\dot{z}(t)+\left(k-{\bar{c}}_{1}\right)z(t)=F_{\mathrm{drv}}(t)$$

Here, Equation (17) can be used to describe the average impact of interaction force on the transfer function of the cantilever within one cycle. *H*_c,T-S_ can be formulated as Equation (18),

(18) $$H_{c,T\text{-}S}=\frac{C_{c}}{ms^{2}+\left(c+{\bar{c}}_{2}\right)s+\left(k-{\bar{c}}_{1}\right)}$$

where *C*_c_ is a non-negative coefficient used to represent the numerical amplification when transmitted from force to displacements. According to Equation (18), the actual resonant angular frequency due to contact and the actual amplitude of TM-AFM can be derived as shown in Equation (19).

(19) $$\left\{\begin{array}{l} \omega_{\mathrm{act}}=\sqrt{\frac{\left(k-{\bar{c}}_{1}\right)}{m}}\\ A_{\mathrm{act}}=A_{0}\frac{\omega_{\mathrm{act}}}{\omega }\sin\varphi \end{array}\right.$$

where *ω*_act_ is the actual resonant angular frequency; *A*_act_ is defined as the actual amplitude of the cantilever. According to Equation (12), Equation (13) and Equation (16), we can observe that *A*_act_ would be equal to *A*_sp_ in the absence of the energy dissipation. However, if the energy dissipation is present, the discrepancy between *A*_act_ and *A*_sp_ results in a fake height signal for cantilever, which will finally mislead PI controller during scan. It is crucial to eliminate such errors according to the variations in sin*φ*. Afterwards, to clarify the impacts of the phase lag to the stability of system, it would be necessary to separately discuss in the following two cases:

(1) *φ* belongs to 0–90°

In this condition, ${\bar{c}}_{1}$ would be negative since cos*φ* ≥ 0. As illustrated by the dotted spectrum in Figure 2c, Equation (19) reveals an increase in the equivalent elasticity of the cantilever, resulting in a shift in the resonant frequency towards higher values and a concurrent decrease in resonance peak. Furthermore, due to the influence of the energy dissipation, an increase in the equivalent damping will lead to a rise in amplitude, as illustrated by the amber curve in Figure 2c. Based on the equivalent model of the cantilever in Equation (18), the close loop transfer function of the system can be transformed as Equation (20),

(20) $$TF_{\mathrm{sys},\;\mathrm{close}}=\frac{K_{E\text{-}D}\omega_{z}^{2}\left(K_{p}s+K_{i}\right)}{s(s^{2}+(\omega_{z}/Q_{z})s+\omega_{z}^{2})\left[ms^{2}+\left(c+{\bar{c}}_{2}\right)s+\left(k-{\bar{c}}_{1}\right)\right]+C_{c}K_{E\text{-}D}\omega_{z}^{2}\left(K_{p}s+K_{i}\right)}$$

From Equation (14), we noticed that energy dissipation is only related to *c*_2_(*t*), so the coefficient ${\bar{c}}_{1}$ in Equation (20) can be neglected to simplify analysis. To assess the variation in system stability, we first define the root of system at critical oscillation as *S*_1_ = *a*_0_*j*. When energy dissipation presents, the variation in *S*_1_ along the imaginary axis can be ignored because *F*_int_(*t*) is much lower than the driving force. Equation (21) can be applied for calculating the PI gains,

(21) $$S_{1}(S_{1}^{2}+\frac{\omega_{z}}{Q_{z}}S_{1}+\omega_{z}^{2})\left[mS_{1}^{2}+\left(c+{\bar{c}}_{2}\right)S_{1}+k\right]+C_{c}K_{E\text{-}D}\omega_{z}^{2}\left[\left(K_{pc}+\Delta K_{p}\right)S_{1}+\left(K_{ic}+\Delta K_{i}\right)\right]=0$$

where *K_pc_* and *K_ic_* refer to the PI gains at critical oscillation without energy dissipation, and Δ*K_p_* and Δ*K_i_* are the adjustments of *p* gain and *I* gain. Δ*K_p_* and Δ*K_i_* can be derived as Equation (22),

(22) $$\left\{\begin{array}{l} \Delta K_{p}=\frac{c_{z}a_{0}^{2}}{C_{c}}{\bar{c}}_{2}\\ \Delta K_{i}=\frac{a_{0}^{2}\left(\omega_{z}^{2}-a_{0}^{2}\right)}{C_{c}K_{E\text{-}D}\omega_{z}^{2}}{\bar{c}}_{2} \end{array}\right.$$

It is clear that if the damp of cantilever decreases, Δ*K_p_* would be negative, so *K_p_* should be lower to avoid system oscillation. The sign of Δ*K_i_* would be determined by the resonant frequency of the Z-scanner.

(2) *φ* belongs to 90–180°

For the condition that *φ* falls in range of 90–180°, a similar trend is observed: with the increase in the equivalent damping, the increase in energy dissipation would result in the decrease in system stability, and the phase lag will increase towards 90°. It becomes evident that the deviation between sin*φ* and sin*φ*_0_ can quantitively express the impact of the energy dissipation to PI gains in critical oscillation.

**Figure 3:**
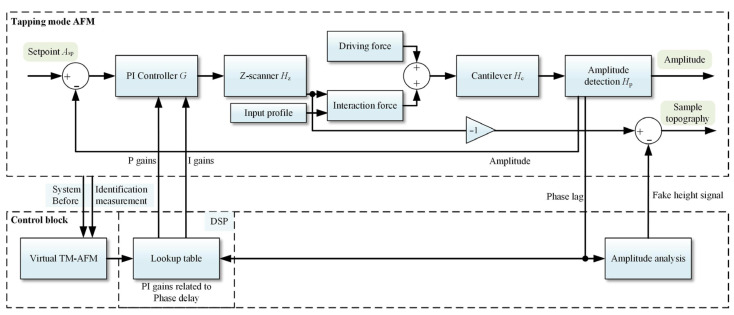
Diagram of the proposed automatic PI control strategy for TM-AFM.

According to the above investigations, it is essential to implement dynamic control if energy dissipation fluctuates during a single scan or among consecutive measurements. It is necessary for two reasons: firstly, to compensate for fake height signals, and secondly, to promptly adjust the PI gains to prevent system oscillation. Consequently, a dynamic control strategy for TM-AFM is proposed, as depicted in Figure 3. Unlike fixed PI control methods, the relationship between the critical PI gains and the phase lag would be numerically determined using the virtual TM-AFM based on SIMULINK and summarized in a lookup table before measurement. During scanning, the Digital Signal Processing (DSP) unit dynamically sends the optimal PI gains to the measurement system and the height compensation module based on predefined lookup table. Subsequently, the height error is determined based on variations in phase lag and compensated for real time to enhance the accuracy of measurement.

## 3. Simulations

### 3.1. System Virtualization

**Figure 4:**
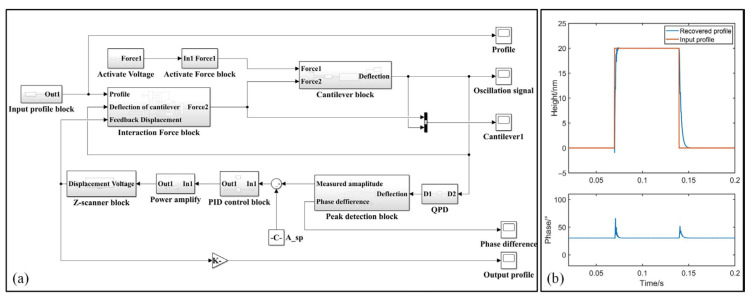
Tapping Mode line-scan virtual framework applied in this work. (**a**) Diagram of the proposed line-scan virtual framework based on SIMULINK. (**b**) Simulations of a 20 nm standard step using the proposed line-scan virtual framework.

To solidify our investigation, a tapping mode virtual AFM framework is established based on SIMULINK in MATLAB R2018a to ensure simulations as close as possible to the real one. As described in Figure 4a, we developed the modules of each individual block and integrated them to simulate a line scan of TM-AFM [31]. The system includes sub-modules such as cantilever block, input profile block, interaction force block, amplitude detection block and PI control block, etc. In the interaction force block, the design of the adhesion force models between the tip and the sample has been categorized as the DMT model, JKR model and M-D model according to the adhesion level of interaction force [31], allowing for force analysis to be completed under all distinct conditions.

Figure 4b presents the simulations of a 20 nm step based on our virtual TM-AFM. In this simulation, non-energy-dissipation presents between the tip and the sample. The morphology of the step is well-restored with the profiles closely matching the input step height. Through the control of the driving force, the free resonance amplitude is set as 60 nm, and the setpoint amplitude is preset to half of the free resonance amplitude. The phase lag stabilizes at 30° in Figure 4b, where sin*φ* is equal to 0.5. The measurement error in the step edge can be clearly observed according to the variation in phase lag.

### 3.2. Phase Lag Analysis

The relationship between the phase lag and the interaction force is simulated based on the proposed virtual system. Since the adhesion force is one predominant sample-related factor to energy dissipation, the surface energy is applied for simulation of the level of energy dissipation. The free oscillation amplitude of cantilever *A*_0_ is selected as 60 nm to simulate actual conditions. The preliminary settings of the internal gains of the proposed virtual system are shown in Table 1, where the features of cantilever are simulated based on TESPA-R3 from Bruker Co. (Billerica, MA, USA). *E*_tip_ and *R*_tip_ are defined as the elastic modulus and radius of the tip, respectively.

**Figure 5:**
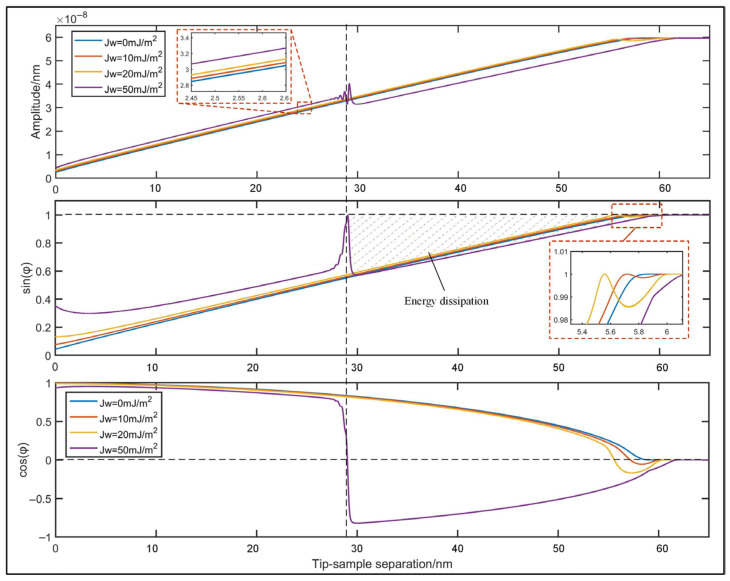
Relationship between phase lag and tip-sample separation during approaching under different surface energies.

The amplitude and the phase lag during approach are previously analyzed based on our virtual system. The elastic modulus of the sample is set as 100 MPa, and a set of surface energies ranging from 0 mJ/m^2^ to 50 mJ/m^2^ are selected to quantify adhesion force. As given by the approaching simulation resulted in Figure 5, with the decrease in tip–sample separation, cos*φ* will transit from negative to positive, while sin*φ* and *A*_act_ will roughly decrease. With the increase in surface energy, more approaching movement is required for local maximum of sin*φ*, thus resulting in the delay of phase lag. This phenomenon is quite consistent with the theoretical analysis. It is also noticed that sin*φ* will reach local maximum at cos*φ* = 0, and this local maximum will move to the left with the increase in surface energy. It can be inferred that the dashed area in Figure 5 corresponds to the energy dissipation. The stability of the system under such circumstances is quite weak. Therefore, it would be advisable to reduce the setpoint to escape from this region to ensure measurement stability.

**Figure 6:**
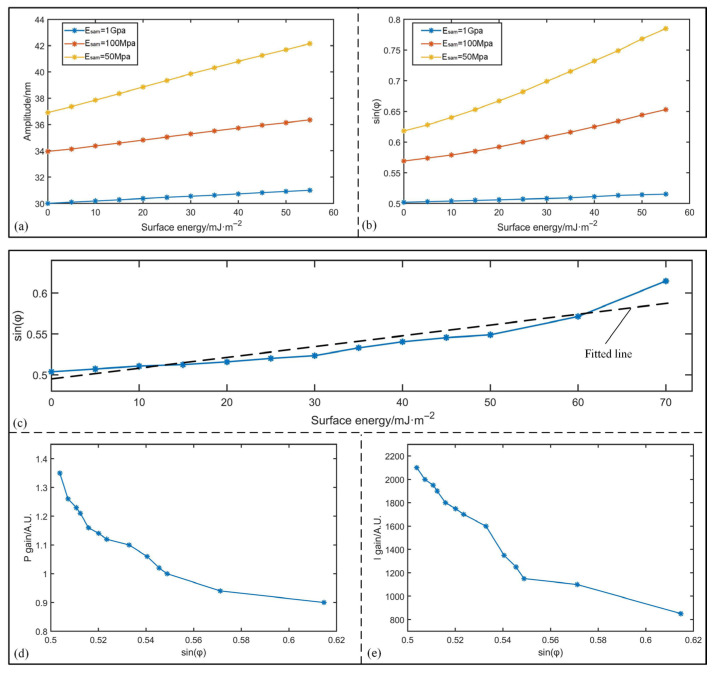
Simulations of system stability and phase lag under different surface energy. (**a**) Actual amplitudes of cantilever under different surface energies. (**b**) Phase lags of cantilever under different surface energies. (**c**) Relationship between phase lag and surface energy. (**d**) *p* gain at critical oscillation. (**e**) *I* gain at critical oscillation.

Moreover, the impacts of the adhesion force with different elastic modulus of the sample are further analyzed for TM-AFM. The surface energy is selected from 0 mJ/m^2^ to 50 mJ/m^2^, and three kinds of elastic modulus *E*_sam_ are also involved, which are 50 MPa, 100 MPa and 1 GPa, respectively. The distance between the sample and the initial balance position of the cantilever is set as 30 nm. When the PI controller is enabled and the cantilever reaches a stable state, the actual amplitude can be equal to the movements of the Z-scanner. Figure 6a,b give the variations in phase lag as well as actual amplitude. It is evident that both sin*φ* and *A*_act_ increase as surface energy rises. When *E*_sp_ is relatively higher and surface energy is lower, sin*φ* approximates closely to *A*_act_/*A*_0_, and the actual amplitude approximates *A*_act_. This is particularly evident when *E*_sam_ = 1 GPa and surface energy is 0 mJ/m^2^, where sin*φ* is less than 0.51 and the actual amplitude is merely 30.02 nm. Therefore, besides the surface energy, we can deduce that the stiffness of the sample determines the contact area and subsequently influences energy dissipation.

Furthermore, the system stability under different surface energies is investigated. Fixing the elastic modulus as *E*_sam_ = 1 GPa, Figure 6c–e present the relationship between the PI gains at critical oscillation and variation in phase lag under different levels of surface energies. The dotted line in Figure 6c refers to the fitted result of sin*φ* with slope of 1.05 × 10^−3^ m^2^/mJ. As shown in Figure 6d–e, both *p* gain and *I* gain will decrease with increased sin*φ*. When sin*φ* changes from 0.506 to 0.615, the variation in *I* gain even reaches 1250, indicating the decrease in system stability. The ratio of PI gains at sin*φ* = 0.615 compared to the ones at sin*φ* = 0.506 are calculated as 0.67 and 0.41, respectively. According to the above analysis, the effectiveness of our research was verified based on the designed virtual framework.

## 4. Experiments

**Figure 7:**
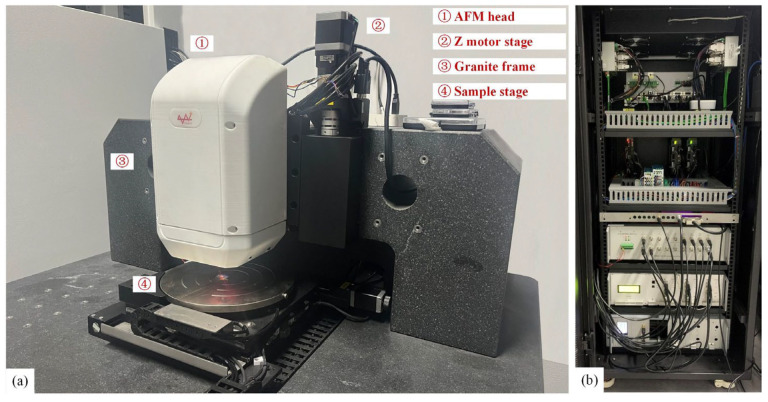
Overview of the experiment AFM system. (**a**) Main structure. (**b**) Electronics system.

Figure 7 gives the overview of the experiment setup, which is a laboratory-developed AFM system with an 8-inch sample stage. This system employs a tip-scanning scheme, of which the scanning range is 100 × 100 × 15 µm^3^, and the noise floor is about 0.05 nm. The type of cantilever used for measurement is TESPA-R3 from Bruker Co. A system identification of the experimental equipment was initially carried out through a frequency scan, resulting in the transfer functions of both the *Z*-scanner and cantilever, as formulated in Equation (23),

(23) $$\left\{\begin{array}{l} H_{c}(s)=\frac{7.29\times 10^{10}}{s^{2}+1714s+7.29\times 10^{10}}\\ H_{z}(s)=\frac{6.46\times 10^{2}}{s^{2}+1410s+1.96\times 10^{8}} \end{array}\right.$$

The corresponding gains in each part of AFM were also retrieved and added on the SIMULINK blocks. After that, by utilizing the virtual framework outlined in Section 3.1, the PI gains of the system during critical oscillation were investigated and summarized in the lookup table provided in Table 2. This lookup table allows the system to operate with an appropriate response speed while maintaining a stable state with update rate of 500 Hz. The gains employed for scanning will be 0.8 times of those listed in Table 2 to guarantee measurement stability. The range of phase lag variation is considered between 0° and 32° to accommodate the actual measurement environment. The lateral scan rate of the AFM is preset as 0.5 Hz, and the scan pixel is set as 256 × 256 pixels^2^.

### 4.1. Standard Height Grid Measurement

To evaluate the performance of the proposed strategy, a calibration nanogrid (BudgetSensors Co., Ltd., Sofia, Bulgaria, HS-20MG, certified height 19.5 ± 0.8 nm) was scanned using both automatic and fixed PI configurations. The grid under test is fabricated though featuring silicon dioxide structure arrays on a silicon substrate; therefore, there will be a difference in phase between the top and the bottom of grid.

The fixed PI strategy maintains PI gains at *K_p_* = 10 and *K_i_* = 1600 throughout imaging. The phase lag map of the sample was initially characterized using fixed PI gains with the detected voltage of the cantilever’s amplitude set as 1 V at free oscillation and the setpoint voltage set as 0.5 V. The initial time delay between the driving signal of the cantilever and the input of the PID controller is recorded as 30° in the experiment.

**Figure 8:**
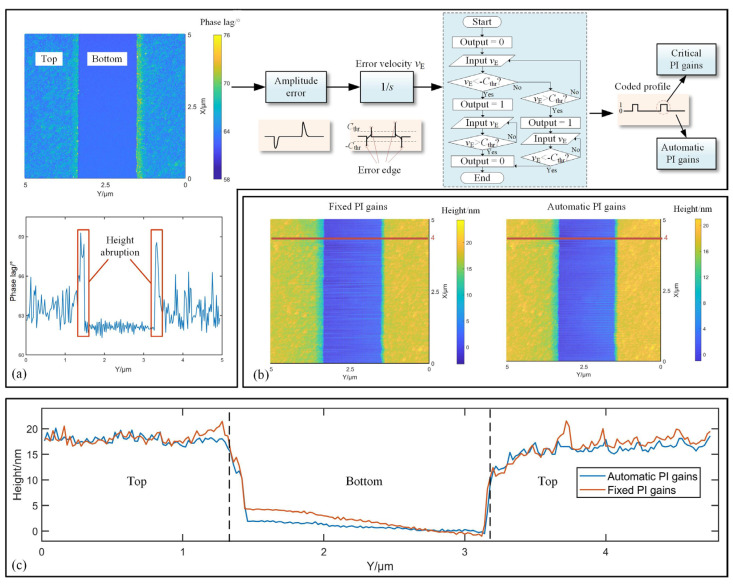
Measurement results of grid. (**a**) Retrieved phase lag map of grid as well as the strategy to enhance response speed of PI controller in step edge. (**b**) Measurement comparisons of height axial views between using fixed PI gains and the proposed method. (**c**) Comparison of profiles along the line at X = 4 µm from Figure 8b.

Figure 8 quantifies the phase lag map as well as the topography of the measured region. The processing procedure of the grid significantly influences the phase lag discrepancies between the bottom and the top areas. Specifically, Figure 8a shows that the phase lag at the bottom varies between 61.5° and 62.6°, whereas the phase lag on the top ranges from 62° to 68°. Furthermore, it is notable that significant phase lag arises in areas of step edge. It would be quite essential to opt for larger PI gains in the step edge, rather than dynamic ones, to improve tracking accuracy. To address this issue, the diagram presented in the right section of Figure 8a is utilized. Defining the amplitude error as the deviation between the retrieved amplitude curve and the setpoint amplitude, by differentiating the error signal, we can obtain both the maximum and minimum vertical speeds of the Z-scanner at each step edge. Empirically, the time it takes to pass through each step edge is much less than 1/5 of the scanning time for each line, so the vertical speed of the Z-scanner in step edge is usually higher than the ratio of the sample height to this transition time. Therefore, a set of maximum and minimum speed position in one step edge can be searched by comparing the real-time vertical speed with a given threshold. Related to scanning speed, scanning range and sample height, this threshold can be defined as Equation (24),

(24) $$C_{\mathrm{Thr}}=5\left|\frac{H_{\mathrm{sam}}v_{\mathrm{scan}}}{L_{\mathrm{scan}}}\right|$$

where *C*_Thr_ is defined as the threshold, *H*_sam_ is the height of sample, *v*_scan_ is the velocity of X-scanner and *L*_scan_ refers to the lateral scanning range along X-axis. Subsequently, the area between each pair of extreme points is set as “1”, while the area outside these pairs is regarded as “0”. The error signal is then converted into a binary form, where the regions encoded as “1” will undergo adjustment of PI gains for critical oscillations.

Figure 8b presents measurement comparisons through first-order plane fit between the proposed strategy and the traditional fixed PI methods. It is evident that the structure measured using dynamic PI gains yields comparable results to those obtained with fixed gains. For instance, considering the profiles at X = 4 µm in Figure 8c, the heights of the grid retrieved by the proposed method and the method using fixed gains are 19.6 nm and 19.8 nm, respectively. The height standard deviations for the top region, using the method proposed in this paper and the one with fixed PI gains, are 0.13 nm and 0.28 nm. Based on the results above, it appears that the height deviation obtained using the proposed method is relatively smaller than that achieved with fixed PI gains, indicating that employing dynamic PI gains not only effectively mitigates the risk of oscillation but also enhances measurement precision.

### 4.2. Half-Coated Silicon Measurement

**Figure 9:**
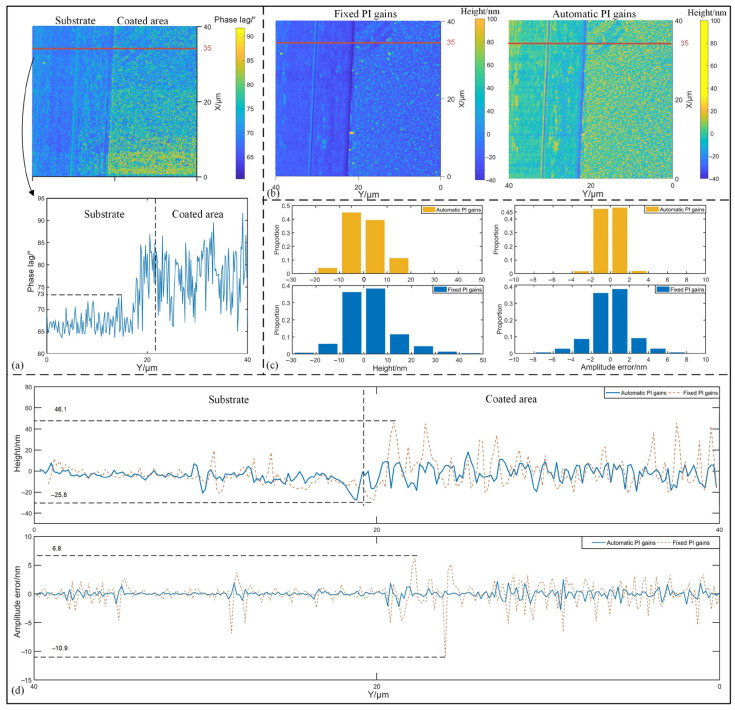
Measurement result of the metal coated sample. (**a**) Retrieved phase lag map from coated sample as well as profiles along the line at X = 35 µm. (**b**) Measurement comparisons between the proposed method and using fixed PI gains. (**c**) Bar graph views of measured height and amplitude error for the proposed method and using fixed PI gains. (**d**) Comparisons of the profiles and amplitude errors at X = 35 µm.

To further validate the effectiveness of the proposed strategy, a silicon-based sample coated with a metal film of several nanometers thickness is measured. Half of the measurement region was processed to expose the silicon substrate for comparison. The time delay between the driving signal of the cantilever and the input of PID controller is recorded as 30° in this experiment. Figure 9a displays the phase lag map of the sample, where the substrate and the coated areas can be clearly distinguished based on phase lag variations. Taking the profile at X = 35 µm as an example, the irregularity of the coating is reflected by phase lag fluctuations ranging from 63° to 92° at the coated positions. Such irregularity may significantly impact measurement accuracy.

Prior to the experiment, a pre-scan was conducted for protecting the AFM head. It was found that due to the energy dissipation, the surface structure could not be effectively retrieved using higher PI gains. The optimal fixed PI gains were preset as *K_p_* = 5.1 and *K_i_* = 700.

Figure 9b displays measurement results were obtained using both the proposed strategy and the fixed PI method. Both of these two methods are capable of measuring the sample structures in alignment with the phase map depicted in Figure 9a. However, it is evident that the measurement deviations using fixed PI gains are relatively higher compared to those achieved with the auto-tuning strategy. Figure 9c gives the bar graph comparisons of both measurement height and PI gains, respectively. It can be observed that 85% of the measurement results obtained by the proposed method fall within the range of ±10 nm, whereas the measurements using fixed PI gains account for approximately 75% within this range, even with the maximum measurement result reaching 50 nm. Similar results are evident in the results of the amplitude errors, where the proportion of amplitude errors within ±2 nm exceeds 90% for the proposed method, while that of the fixed PI gains is less than 80%. More clearly, the profile retrieved using fixed PI gains in Figure 9d shows fluctuations within the coated area, ranging from −25.8 nm to 46.1 nm. This is because the controller with fixed gains may not be suitable throughout the entire measurement. Similarly, from the comparison of amplitude error, it is noticed that the maximum error using fixed PI gains reaches ±10 nm, whereas the ones with automatic PI gains are in range of ±2.2 nm. In summary of experiments, the stability of the system can be improved through the dynamic control strategy of PI gains.

### 4.3. Silicon-Base Photoresist Substrate Measurement

**Figure 10:**
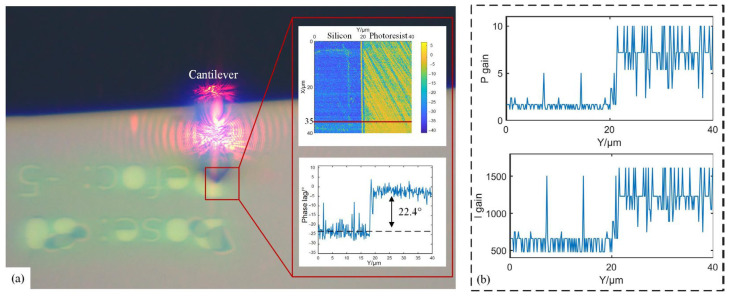
Phase analysis of the sample. (**a**) Photograph of the sample as well as retrieved phase lag. (**b**) Automatic *P* gains as well as *I* gains using the proposed strategy for the profile in Figure 10a.

**Figure 11:**
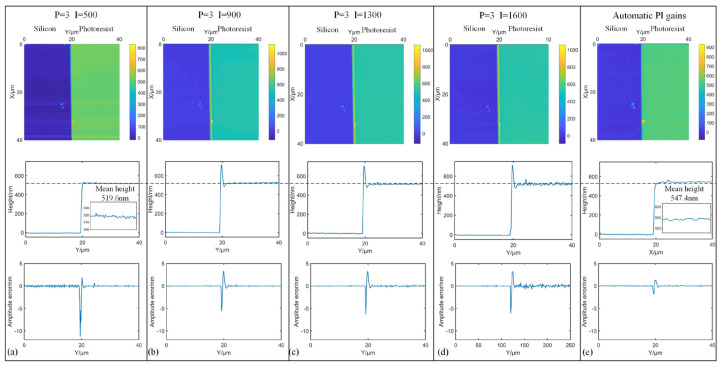
Measurement comparisons under different selections of PI gains. (**a**) *K_p_* = 3, *K_i_* =500. (**b**) *K_p_* = 3, *K_i_* = 900. (**c**) *K_p_* = 3, *K_i_* = 1300. (**d**) *K_p_* = 3, *K_i_* = 1600. (**e**) Automatic PI gains.

**Figure 12:**
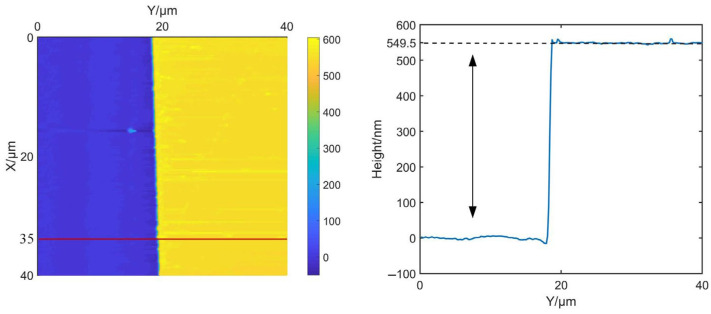
Measurement of the sample in contact mode using a commercial AFM.

Different from the experiments of thin films in Section 4.2, the proposed method is further applied to a silicon-base photoresist substrate sample with a relative height of several hundred nanometers. As illustrated in the snapshot of the measured sample in Figure 10, the alphabet-patterned regions correspond to silicon, while the remaining areas represent the photoresist substrate. The time delay between the driving signal of cantilever and the input of PID controller is recorded as −60° in this experiment. A pre-scan was conducted using PI gains of *K_p_* = 3 and *K_i_* = 500 to generate the phase lag map. Notably, significant phase lag differences are observed between the two material surfaces due to the variation in adhesion forces. Taking the profile at X = 35 µm as example, a phase lag variation between two materials was recorded as 22.4°. As shown in Figure 11, to investigate the impacts of adhesion forces on the optimal PI gains, a series of measurements are performed by fixing *p* gain at 3 while varying *I* gains from 500 to 1600, with results compared with the proposed automatic PI control method. It is clear that the micro structure can be recovered across all *I* gains, but overshoot emerged in the transition zones between two materials when *I* gain exceeded 500. In addition, while lower *I* gain can avoid overshoot and reduce amplitude errors for photoresist, it will impact the response speed and then increase amplitude errors of silicon. The optimal *I* gain is determined to be 1600 for silicon and 500 for photoresist, which indicates that fixed PI gains are inappropriate for materials with significant difference in adhesion force. Conversely, through the auto-tuning of PI controller, the proposed method successfully avoids overshoot and maintains lower amplitude errors of both silicon and photoresist. By calculating the mean values of the base and the top, the height of the pattern in Figure 10 can be retrieved as the relative difference. The measured height of the sample using fixed PI gains is 519.6 nm. However, based on the proposed strategy, the mean height of the sample is corrected to 547.4 nm, where there is a 5.4% improvement in the height of the sample. In addition, the contact mode in AFM is immune to the energy dissipation; therefore, the same region in the sample was also measured in contact mode using a commercial AFM (Dimension ICON, Bruker Co., Billerica, MA, USA). As shown in Figure 12, the measured height of the profile along the line at X = 35 µm is 549.5 nm. This result exhibits the agreement with the measurements obtained using the proposed automatic strategy. Through analysis of phase lag, the effective compensation is made for height errors caused by adhesion force variations.

## 5. Conclusions

This paper reports a novel dynamic PID control method tailored for TM-AFM. Based on the principle of energy dissipation, we conduct an in-depth analysis of the relationship between the phase lag of cantilever and system stability. Subsequently, we successfully derive an automatic tuning strategy for PI gains by means of virtual AFM framework. Distinct from other approaches, our method possesses the capability to automatically adjust PI gains in response to changes in phase lag, thereby effectively ensuring measurement accuracy while preventing system oscillation. The efficacy of our method has been rigorously verified through both simulations and experiments. Our proposed method holds significant industrial application potential, particularly in the microstructural characterization of multi-material samples.

## Figures and Tables

**Table 1:** Mechanic parameters of the cantilever and the Z-scanner.

Parameter	Value	Parameter	Value
*m*	2.0 × 10^−11^ kg	*c*_z_	2.1 × 10^−5^
*c*	2.8 × 10^−8^	*k*_z_	20 N/m
*k*	26 N/m	*E*_tip_	130 GPa
*m*_z_	2 × 10^−3^ kg	*R*_tip_	10 nm

**Table 2:** Lookup table of PI gains at critical oscillation related with phase difference.

Interval of Phase Lag Variation	*p* Gain (*K_p_*)	*I* Gain (*K_i_*)
0–4°	10	1610
4–8°	7.2	1230
8–12°	5.4	1050
12–16°	3.4	890
16–20°	2.6	820
20–24°	2.4	750
24–28°	1.7	660
28–32°	1.2	480

## Data Availability

The data presented in this study are available on request from the corresponding author.

## Abbreviations

The following abbreviations are used in this manuscript:

TM-AFM	Tapping Mode Atomic Force Microscopy
SPM	Scanning Probe Microscopy
STM	Scanning Tunneling Microscopy
SNOM	Scanning Near-Field Optical Microscopy
AFM	Atomic Force Microscopy
PID	Proportional Integral-Derivative
AI	Artificial Intelligence
RMS-DC	Root Mean Square to Direct Current
LIA	Lock-in Amplifier
